# Dynamic PET/CT measurements of induced positron activity in a prostate cancer patient after 50-MV photon radiation therapy

**DOI:** 10.1186/2191-219X-3-6

**Published:** 2013-01-23

**Authors:** Sara Janek Strååt, Hans Jacobsson, Marilyn E Noz, Björn Andreassen, Ingemar Näslund, Cathrine Jonsson

**Affiliations:** 1 Department of Medical Radiation Physics, Karolinska Institutet and Stockholm University, Stockholm, PO Box 260, SE-171 76, Sweden; 2 Department of Molecular Medicine and Surgery, Karolinska Institutet, Stockholm, SE-171 76, Sweden; 3 Department of Radiology, School of Medicine, New York University, New York, NY, 10016, USA; 4 Department of Oncology and Pathology, Karolinska Institutet, Stockholm, SE-171 76, Sweden; 5 Department of Medical Physics, Section of Imaging Physics, Karolinska University Hospital, Stockholm, SE-171 76, Sweden

**Keywords:** High-energy photons, PET, Induced tissue activity, Photonuclear reactions, Radiation therapy, Treatment beam verification.

## Abstract

**Background:**

The purpose of this work was to reveal the research interest value of positron emission tomography (PET) imaging in visualizing the induced tissue activity post high-energy photon radiation treatment. More specifically, the focus was on the possibility of retrieving data such as tissue composition and physical half-lives from dynamic PET acquisitions, as positron-emitting radionuclides such as ^15^O, ^11^C, and ^13^N are produced *in vivo* during radiation treatment with high-energy photons (>15 MeV). The type, amount, and distribution of induced positron-emitting radionuclides depend on the irradiated tissue cross section, the photon spectrum, and the possible perfusion-driven washout.

**Methods:**

A 62-year-old man diagnosed with prostate cancer was referred for palliative radiation treatment of the pelvis minor. A total dose of 8 Gy was given using high-energy photon beams (50 MV) with a racetrack microtron, and 7 min after the end of irradiation, the patient was positioned in a PET/computed tomography (CT) camera, and a list-mode acquisition was performed for 30 min. Two volumes of interests (VOIs) were positioned on the dynamic PET/CT images, one in the urinary bladder and the other in the subcutaneous fat. Analysis of the measured relative count rate was performed in order to compute the tissue compositions and physical half-lives in the two regions.

**Results:**

Dynamic analysis from the two VOIs showed that the decay constants of activated oxygen and carbon could be deduced. Calculation of tissue composition from analyzing the VOI containing subcutaneous fat only moderately agreed with that of the tabulated International Commission on Radiation Units & Measurements (ICRU) data of the adipose tissue. However, the same analysis for the bladder showed a good agreement with that of the tabulated ICRU data.

**Conclusions:**

PET can be used in visualizing the induced activity post high-energy photon radiation treatment. Despite the very low count rate in this specific application, wherein 7 min after treatment was about 5% of that of a standard ^18^F-FDG PET scan, the distribution of activated tissue elements (^15^O and ^11^C) could be calculated from the dynamic PET data. One possible future application of this method could possibly be to measure and determine the tumor tissue composition in order to identify any hypoxic or necrotic region, which is information that can be used in the ongoing therapy planning process.

**Trial registration:**

The official name of the trial committee of this study is ‘Regionala etikprövningsnämnden i Stockholm’ (FE 289, Stockholm, SE-17177, Sweden). The unique identifying number is 2011/1789-31/2.

## Background

Georg de Hevesy became the father of nuclear medicine when he formulated the famous ‘Tracer Principle’ in 1913 (G Jr. de Hevesy, personal communication) [1]. This means that by the administration of minute amounts of a chemical compound labeled with a proper radionuclide, it is possible to study functional mechanisms in living plants, animals, or humans without interfering with their functional properties. Diagnostic nuclear medicine is usually based on the administration of a radiopharmaceutical which undergoes a biological or physiological process in the body that can be depicted and analyzed.

During external beam radiation therapy with high-energy photons (>15 MeV), short-lived positron-emitting radionuclides are generated in the normal tissues through the processes of photonuclear reactions. For ^12^C, ^14^N, and ^16^O in the living tissue, the photoneutron reaction, denoted as (*γ*, *n*), has a threshold energy of about 15 to 18 MeV [2-4]. The process leads to the positron-emitting radionuclides ^11^C, ^13^N, and ^15^O with physical half-lives of 20, 10, and 2 min, respectively. The amount of positron emitters produced per unit volume will depend on the shape of the photoneutron cross section for the specific element and on the tissue density as well as the energy spectrum of the incident photon beam.

The aim of this report is to highlight a way of producing radionuclides in a patient, thus providing information that may be complementary to that from traditional nuclear medicine. Therefore, we describe a positron emission tomography (PET) study in a male patient after high-energy photon treatment of the pelvis minor because of the local spread of prostate cancer. The examination was done as an extension of earlier work described in [5] which was an attempt to verify the dose location of the radiation beams. Analysis of the series of consecutive PET acquisitions initiated at the end of the treatment showed a cascade of decaying radionuclides with different decay patterns depending on the composition of the various tissues irradiated. The amount of information was more than one could achieve on the basis of a regular nuclear medicine examination. As for today, a total number of four racetrack microtrons using 50-MV photons are in clinical use in China, and more units are being installed (E Jöreskog, personal communication). Consequently, this or a similar technique based on nuclear activation may have future applications.

## Methods

### Patient

A 61-year-old man was diagnosed with prostate cancer with extensive local growth. Bone scintigraphy also showed an uptake in the right os ischii and os pubis due to metastases.

### Radiation treatment and beam setup

**Figure 1:**
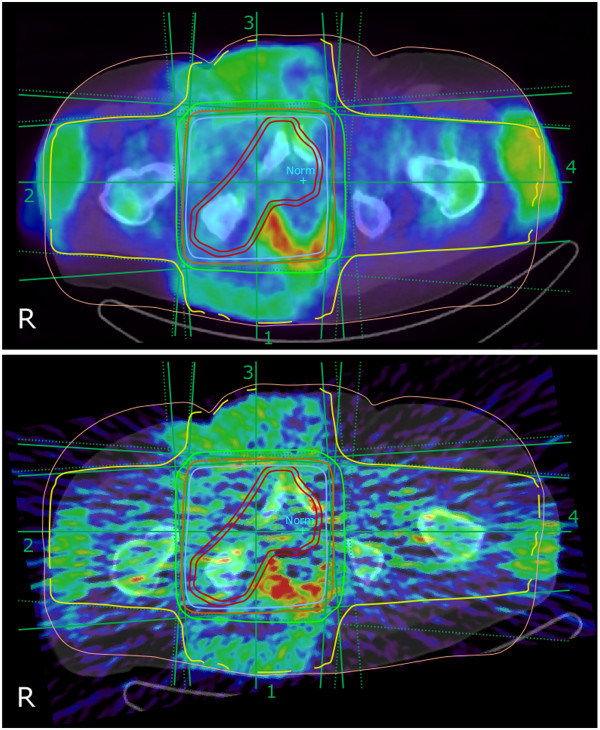
**Transaxial overlaid 50-MV radiation treatment plan on PET/CT (0 to 30 min) image.** This is for three-dimensional ordered subset expectation minimization (3D-OSEM, upper) and filtered back projection (FBP, lower) reconstruction. The treatment, here shown for plane section through the normalization point (Norm), is a four-field box technique with the target dose delivery of 8 Gy in total divided equally between the four beam portals. The isodose curves displayed cover the ranges 35% (yellow), 65% (green), 85% (orange), and 95% (blue) of the total dose. The two red curves depict the interactively defined clinical target volume (inner line) and planning target volume (outer line). The gross tumor volume is not depicted but includes the prostate together with the metastasis of the right os ischii and the medial portion of the right os pubis.

The patient was scheduled for palliative radiation treatment consisting of five fractions delivered in a span of 1 week. The first four fractions were delivered to the patient using 18-MV photons. The last fraction was delivered using 50-MV scanned photon beams (Racetrack Microtron MM50 Scanditronix, IBA, Uppsala, Sweden), giving a total dose of 8 Gy. For the purpose of this and another patient study, the racetrack microtron had to be restored to its original clinical state; thus, only one of the five fractions was delivered using 50-MV photons. In Figure 1, the 50-MV photon treatment plan is shown (together with the PET/computed tomography (CT) image) for selected transaxial and coronal planes. The target included the primary tumor and the metastases of the right pelvic bones. The target dose delivery was divided equally between four beams in the following order: (1) posterior-anterior, (2) right, (3) anterior-posterior, and (4) left. The total irradiation time, including rotation of the gantry, was 6 min and 52 s.

### PET/CT acquisition and patient positioning

The PET/CT scan was performed with a Siemens Biograph TrueV PET/CT scanner (Siemens Medical USA, Knoxville, TN, USA) located at the Nuclear Medicine Department. The time span between the end of the irradiation and the start of the PET/CT scan was approximately 7 min. Patient transport was arranged with a wheelchair. The CT scan was a ‘low-dose attenuation correction CT’ (ACCT) for necessary corrections and an anatomical reference. The PET acquisition was performed in list mode, allowing for the optional selection of frame lengths. In order to eliminate decay correction issues when reconstructing time frames, the isotope information for the acquisition protocol was set to ^68^Ge (half-life, *T*_1/2_ = 271 days). Reconstruction was performed with filtered back projection (FBP) and attenuation-weighted three-dimensional ordered subset expectation minimization (3D-OSEM) (two iterations and eight subsets), creating images with somewhat different characteristics but derived from the same raw data. A 5-mm post-reconstruction Gaussian filter was applied in all reconstructions. All data were corrected for random coincidences, dead time, scatter, and attenuation. Reconstructed dynamic series were created using a protocol consisting of 15 frames of 2 min each. In addition, a summation image was created using the complete acquired dataset, i.e., 30 min in duration. The PET study was, thus, performed on clinical indication. After a retrospective application, the regional ethical committee, ‘Regionala etikprövningsnämnden i Stockholm’ (Stockholm, Sweden), declared no objection to the study.

### Image fusion of PET/CT and dose planning CT

The volume co-registration software, Mirada (XD3, Mirada Medical, Oxford UK) [6], was used to align the ACCT to the planning CT. Mirada uses a voxel intensity-based registration algorithm that can be both rigid and deformable. In this study, the deformable registration was used. The fusion was performed by an experienced radiologist interactively moving and rotating the ACCT volume until a satisfactory fit to the planning CT was found. All four image sets (PET, ACCT, planning CT, and treatment plan data) were simultaneously displayed, allowing proper alignment of the ACCT and the planning CT.

### Image analysis

The PET/CT images were interpreted by an experienced radiologist. Two volumes of interest (VOIs) were positioned on the dynamic PET/CT images. VOI 1 was positioned at a region highlighted in the PET images, corresponding to the subcutaneous fat lateral to the left hip joint. VOI 2 was drawn with a certain margin within the urinary bladder as depicted in the CT sections. Image viewing and VOI evaluation was performed using AMIDE software [7,8]. Analysis of the measured relative count rate in the two regions was performed with MATLAB [9]. Due to the fact that the therapy machine and the PET/CT unit were not co-located, any washout of positron emitters in the patient was not measurable from the PET data. In order to obtain such data, preferably in-beam PET measurements need to be performed as indicated by animal and patient studies during ^11^C and ^12^C beam irradiation [10-12]. Thus, decay of biological components was not considered in the analysis. Assuming ^12^C, ^16^O, and ^14^N to be the most dominant in the tissue, the time-dependent measured total count rate (per second; proportional to the activity), *S*(*t*), can be written as the sum of three exponential terms:

(1) $$\begin{array}{c} S\left(t\right)=S_{1}\;\exp\;(-\ln\left(2\right)\times \frac{t}{T_{\frac{1}{2},\;1}})+S_{2}\;\exp(-\ln\left(2\right)\\ \times \frac{t}{T_{\frac{1}{2},\;2}})+S_{3}\exp(-\ln\left(2\right)\times \frac{t}{T_{\frac{1}{2},\;3}})+K\text{,} \end{array}$$

where *S*_1_, *S*_2_, and *S*_3_ represent the count rates from radionuclides with physical half-lives *T*_½,1_, *T*_½,2_, and *T*_½,3_, respectively, at the end of the irradiation (corrected for radioactive decay is 7 min, i.e., the transport time between when the irradiation finished to the start of the PET scan). *K* is a constant. The quantities *S*_1_, *S*_2_, *S*_3_, *T*_½,1_, *T*_½,2_, and *T*_½,3_ are computed by performing a least square fit, which minimizes the sum of the squares of the differences of measured data from Equation 1. A solution was obtained for the following two cases: (i) *S*_1_, *S*_2_, and *S*_3_ were set as unknowns while *T*_½,1_, *T*_½,2_, and *T*_½,3_ were held fixed and set according to the half-lives of ^11^C, ^15^O, and ^13^N, respectively, and (ii) *S*_1_, *S*_2_, *S*_3_, *T*_½,1_, *T*_½,2_, and *T*_½,3_ were all set as unknowns. Thus, the following constraints were set for the two cases (i and ii for Equations 2 and 3, respectively):

(2) $$S_{1},S_{2},\mathrm{and}\;S_{3}\geq 0\text{,}$$

$$\begin{array}{c} T_{\frac{1}{2},1}=T_{\frac{1}{2},11C}=\;20.39\;\min,\\ T_{\frac{1}{2},2}=T_{\frac{1}{2},15O}=\;2.04\;\min,\\ T_{\frac{1}{2},3}=T_{\frac{1}{2},13N}=\;9.97\;\min\text{,} \end{array}$$

and

(3) $$S_{1},S_{2},\mathrm{and}\;S_{3}\geq 0\text{,}$$

$$T_{\frac{1}{2},1},T_{\frac{1}{2},2},\mathrm{and}\;T_{\frac{1}{2},3}\geq 0.$$

In equation 3, *T*_½,1_, *T*_½,2_, and *T*_½,3_ were used to extract the estimated physical half-lives (*T*_½,11C_, *T*_½,15O_, and *T*_½,13N_) of ^11^C, ^15^O, and ^13^N, respectively. The computed quantities *S*_1_, *S*_2_, and *S*_3_ are compared to ^1^H-adjusted^a^ International Commission on Radiation Units & Measurements (ICRU) elemental composition for the urinary bladder (filled) and adipose tissue [13].

## Results and discussion

### Results

#### In vivo visualization of the treatment beams by PET

It could be estimated that the activity concentration in a radiotherapy-treated patient, 7 min after a 5- to 8-Gy treatment, is close to 5% of that of a patient injected with ^18^F-FDG for a standard PET scan. Even if FBP has its strength in being a linear process, there are obvious drawbacks looking at the visual appearance of the images. The resulting images using FBP were noisy and contained pronounced streak artifacts. Therefore, to facilitate outlining of the VOI, images from iterative 3D-OSEM reconstruction were used. In Figure 1, the PET/CT (0 to 30 min) image is shown side by side for both reconstruction methods. Clearly visible in both reconstructions is a high-induced activity seen in the subcutaneous fat where the four beams entered the patient.

#### Dynamic VOI analysis

**Figure 2:**
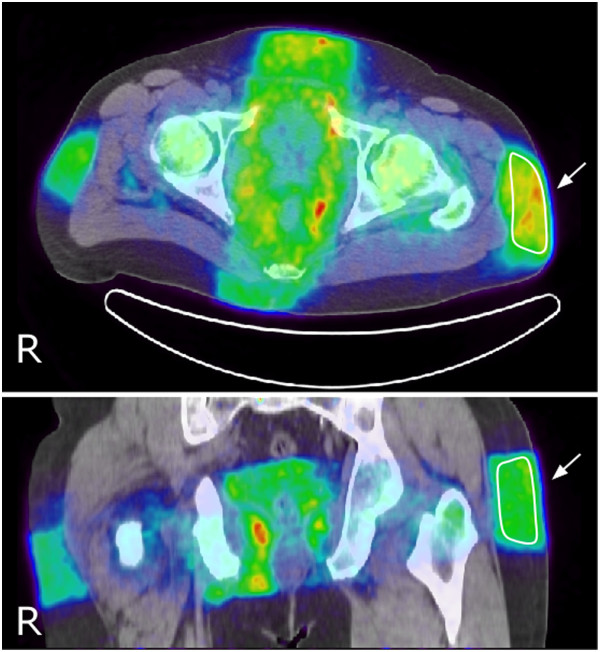
**Transaxial (upper) and coronal (lower) PET/CT (0 to 30 min) sections.** The figure illustrates how VOI 1 (arrow) was drawn on the dynamic PET image in the region of the beam entrance in the subcutaneous fat.

**Figure 3:**
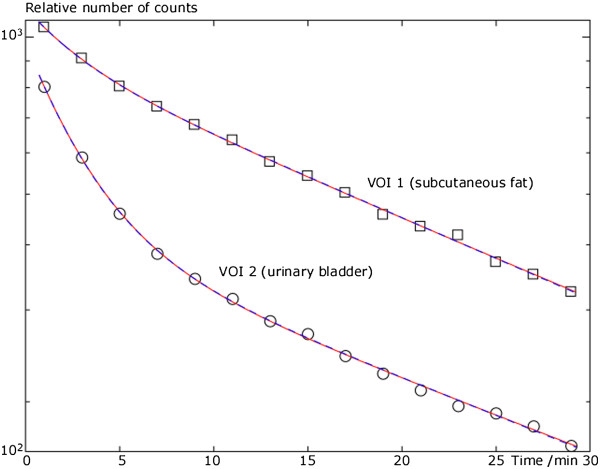
**Relative number of counts for both VOIs as function of time displayed in linear-logarithmic diagram.** Measured data are shown as circles and squares together with the best fit to Equations 2 and 3 represented as a solid red line and a dashed blue line, respectively.

**Figure 4:**
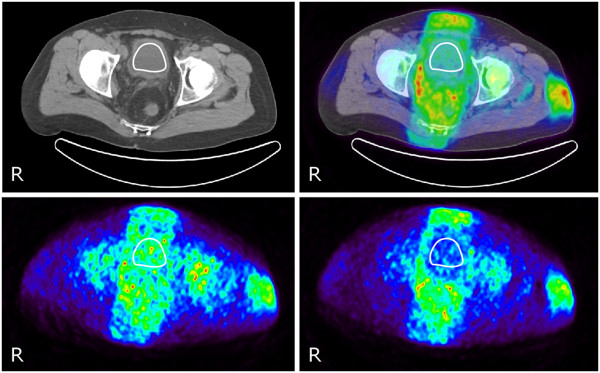
**Transaxial sections of the ACCT (left) and PET/CT (right) (0 to 30 min).** The image shows the VOI drawn in the urinary bladder (upper row). Lower row shows the transaxial section of the PET image acquired from 0 to 2 min (left) and from 25 to 30 min (right) along with the VOI imposed in the urinary bladder. During this time period, the bladder activity representing ^15^O has almost completely declined while the surrounding soft tissue activity, mainly representing ^11^C, is still clearly visible.

1. *VOI 1*. The VOI drawn over the subcutaneous fat on the PET image is presented in Figure 2 for transaxial and coronal planes. The relative number of counts as a function of time for VOI 1 is shown in Figure 3, with the lines representing the best fit to Equations 2 and 3 in solid red and dashed blue, respectively. For Equation 2, it was found that *S*_1_ = 0.32 ± 0.11, *S*_2_ = 0.65 ± 0.21, *S*_3_ = 0.03 ± 0.24, and *K* = 0 representing the relative count rate, with calculated 95% confidence interval, from ^11^C, ^15^O, and ^13^N at the end of irradiation. For Equation 3 instead, it was found that *S*_1_ = 0.33 ± 0.01, *Τ*_1_ = 19.4 ± 1.1 min, *S*_2_ = 0.67 ± 0.11, *Τ*_2_ = 1.99 ± 0.74 min, *S*_3_ = 0, and *K* = 0. The values of *S*_1_, *S*_2_, and *S*_3_ are compared with the ^1^H-adjusted^a^ ICRU elemental compositions for the adipose tissue of adult 2^a^ in [13] which have the following values: 67.7% C, 31.5 O, and 0.8% N.

2. *VOI 2*. The urinary bladder as defined by the CT images is shown in Figure 4; it also shows the sum of the complete dynamic PET study (0 to 30 min). An early image (0 to 2 min) and three added late images (25 to 30 min) from the dynamic PET study are also presented. The count rate in the urinary bladder is high at the beginning of the acquisition but declines to almost zero after 25 min. The relative number of counts as a function of time for VOI 2 is shown in Figure 3, with lines representing the best fit to Equation 2 in solid red and Equation 3 in dashed blue. It was found that the relative count rates, with calculated 95% confidence interval, from ^11^C, ^15^O, and ^13^N at the end of irradiation were *S*_1_ = 0.08 ± 0.00, *S*_2_ = 0.92 ± 0.03, *S*_3_ = 0, and *K* = 0 for Equation 2. Applying the constraints in Equation 3, the computation gave that *S*_1_ = 0.08 ± 0.00, *Τ*_1_ = 20.9 ± 1.6 min, *S*_2_ = 0.92 ± 0.04, *Τ*_2_ = 2.01 ± 0.18 min, *S*_3_ = 0, and *K* = 0. The values of *S*_1_, *S*_2_, and *S*_3_ are compared with the ^1^H-adjusted^a^ ICRU elemental compositions for the urinary bladder (filled) of the adult in [13] which have the following values: 4% ^12^C, 94% ^16^O, 1.7% and ^14^N.

### Discussion

This work focused on the potential use of PET for measuring the *in vivo*-induced tissue activity due to radiation treatment with high-energy photons, which is 50 MV in this case. Since 1972, attempts were already made at our institution using a gamma camera to depict the distribution of the positron-emitting radionuclides produced in patients being irradiated with 42-MV photons from a betatron. The acquired images could clearly verify that the radionuclide distribution coincided with the irradiated regions. However, as the sensitivity of the camera was very low and the output only analogous, no further such attempts were made (L Johansson, personal communication). Other early studies have shown the potential use of tissue activation for the analysis of tumor blood flow in animal studies [14-16]. More recently, by the study of biological washout processes of ion-beam-induced positron emitters, half-lives of various washout components could be measured in animals [10,11] and in patients [12,17]. Furthermore, dose and treatment beam verification during as well as immediately after treatment with ion-beams [18,19] and protons [17,20,21] in patients and for high-energy photons in the animal tissue [5] have been reported.

In this study, measurements of biological washout processes in the patient were not relevant due to the fact that the therapy machine and the PET/CT were not co-located, and most of the radioactivity had been eliminated before the PET examination. Studying half-lives of various washout components, particularly in blood-rich organs such as the lungs and the liver, generally needs activity measurements to be performed during or at least in direct connection to the irradiation [22]. The urine of the bladder, on the other hand, represents an enclosed compartment with a negligible exchange with other tissues. From the CT scan, it was estimated that the urine volume of the bladder at the beginning of PET acquisition was approximately 200 cm^3^. Assuming a urine excretion of about 0.5 ml/min, the dilution of activity in this case is almost negligible. Also in fat, the dynamic portion constitutes only of a very small fraction, and most of the induced PET activity originates from radionuclides that are stationary within the tissue.

VOI analysis of the subcutaneous fat gave a composition that did not completely agree with ICRU-tabulated values for the adipose tissue [13], although the fitted half-lives were found to agree well for both ^11^C and ^15^O. The reason for the restricted agreement in composition may be the variations in the actual composition of the subcutaneous fat of the current patient. According to [13], the adipose tissue is the most variable tissue in the body regarding elemental composition. Water content may vary from 10.9% to 21.0%, and lipid content can range from 62% to 91% [23]. From the analysis of urinary bladder contents (i.e., urine), a high level of oxygen content was found, which is expected as it mainly is composed of water. The calculated half-lives and composition were found to correlate well with tabulated ICRU values [13]. In bone tissues, a high content of ^40^Ca and ^31^P will produce positron-emitting radionuclides when they are irradiated with high-energy photons. However, as the half-life of ^39^Ca is only 0.86 s, this activity will probably never be measurable, and ^30^P that has a half-life of 2.5 min will most likely not be distinguishable from ^15^O.

Upon arrival at the Nuclear Medicine department, the setup and positioning of the patient on the PET/CT couch were done as fast as possible in order to avoid further loss of induced activity. The main focus was to ensure that the radiation treatment volume was covered by the axial field of view of the PET camera. Subsequent reconstructions showed that the patient had become slightly mis-positioned in all three planes. In addition, the regular curved PET/CT couch did not match the flat couch used during the radiation treatment and the CT planning, resulting in a deformed activity distribution toward the outer edges of the patient as seen on the treatment plan overlaid on the PET/CT image. However, the deformable registration compensated for the different couches in the main part of the abdomen where the beams intersect.

Reconstruction with FBP [24-26], which is based on the inverse of the radon transform [27], is fast, robust, linear, and known to yield quantitative results. However, in the low count data such as in this study, the results showed a poor visual image quality, disturbing streak artifacts, and high noise. In order to better visualize the activated tissue and, thereby, the positioning of the VOIs, the iterative reconstruction method, 3D-OSEM [28], was preferred. The images reconstructed by 3D-OSEM lack the streak artifacts and contain no data outside the object. However, further studies are required to assess the quantitative accuracy of the two methods. It might be that the VOIs preferably are outlined using the 3D-OSEM algorithm, while the data from the VOIs preferably should be extracted from the FBP reconstructed data.

## Conclusions

The purpose of this work was to reveal the research interest value of PET imaging in visualizing the induced tissue activity post high-energy photon radiation treatment. Despite the very low count rate, the work demonstrates that the distribution of activated tissue elements (mainly ^15^O and ^11^C) could, for stationary tissues, be calculated from the dynamic PET data. The measurement of the mobile as well as stationary tissue might be possible if the PET/CT unit is located close to the radiation treatment facility as this method would be more sensitive. This has been demonstrated in the case of ions [12,17]. As the radionuclides produced in the patient during photon irradiation all originate from the body tissue (and not from the beam itself), the measured PET activity will, with some corrections, be strictly proportional to the body tissue composition. The idea of being able to measure the true body tissue composition and map anatomical structures is interesting [29] and could have future applications. One possible aspect of this method would be to measure and determine the tumor tissue composition in order to identify any hypoxic or necrotic regions that can be used in further therapy planning process.

## Endnotes

^a^The elemental composition was recalculated from ICRU, where hydrogen (^1^H) - which will not become a positron emitter - has been omitted in the calculation.

## Competing interests

The authors declare that they have no competing interests.

## Authors’ contributions

SJS conceived the study, participated in its design and coordination, carried out the following: irradiations, PET/CT measurements, image reconstructions, image fusion, and image analysis, and drafted the manuscript. HJ participated in PET/CT measurements, carried out interpretation of data, and drafted the manuscript. MEN carried out image reconstructions and image fusion and drafted the manuscript. BA carried out irradiations and PET/CT measurements and participated in image reconstructions. IN carried out the dose plan and irradiations, and participated in PET/CT measurements. CJ conceived the study, participated in its design and coordination, carried out the following: PET/CT measurements, image reconstructions, and interpretation of data, and drafted the manuscript. All authors read and approved the final manuscript.
